# Supplementary material for: Cell-in-the-loop pattern formation with optogenetically emulated cell-to-cell signaling
Source: Nat Commun. 2020 Mar 13;11:1355. doi: 10.1038/s41467-020-15166-3 (PMC7069979; doi:10.1038/s41467-020-15166-3)
Supplement: Supplementary file 1 — Supplementary Information [file 41467_2020_15166_MOESM1_ESM.pdf]

Supplementary Information

Cell-in-the-loop pattern formation with optogenetically emulated cell-to-cell signaling

Perkins et al.

1 Graphical test for bistability

Consider a 2-cell system of mutual inhibition with dynamics (and notation) as given in Section 3.1, in which $u_2(t) = h(v_2(t)) = h(w_1(t))$ and similarly $u_1(t) = h(v_1(t)) = h(w_2(t))$. Now break the loop such that $u_1(t)$ becomes the input and $y(t) = h(w_2(t))$ the output of a the *open-loop system*. The static input-output characteristic for the cascade, given by $y^\dagger = h(T(h(T(u_1^\dagger))))$, is increasing. The points where $u_1^\dagger = h(T(h(T(u_1^\dagger))))$ (i.e., intersections of the static input-output characteristic with a line of slope 1) are the steady states of the corresponding *closed-loop system* in which $u_1(t) = y(t)$ (and we still have $u_2(t) = h(w_1(t))$). We will designate values at such intersections by superscript asterisks (e.g., u_1^*). When cell dynamics are monotone, then the steady state corresponding to u_1^* is stable if the slope of $h(T(h(T(u_1^*))))$ is greater than 1 and unstable if it is less than one. In particular for our setup, this implies that if there is one intersection, the closed-loop system is monostable, and if there are three intersections, the system is bistable, with the homogeneous solution ($u_1^* = u_2^*$) being unstable and the other two stable points corresponding to w_1^* high, w_2^* low, and vice versa [1].

2 Detailed methods

2.1 Agarose pad preparation

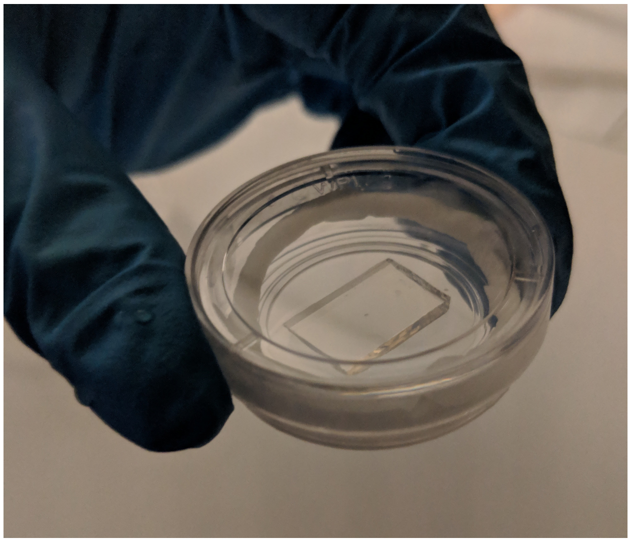

Figure 1: Example agarose pad used in experiments.

Two 2-slide-tall stacks of microscope slides were placed 1 cm apart parallel to each other on top of a single microscope slide. 70 µL of 2% agarose (UltraPure^{TM}Agarose, Invitrogen) and SD medium solution were pipetted between the two stacks. A square 18 mm × 18 mm cover slip was gently placed on the top. The pad was solidified for 1 hr. Immediately before placement under the microscope, the stacks and cover slip were removed and the ends of the pad were sliced off with a scalpel such that the final pad was about 15 mm × 15 mm and level across the top. 3 µL of cell suspension were pipetted in increments of 1 µL onto three separate areas of the pad to ensure that at least one would have the correct density for use in the experiment. The pad was overturned

into a circular tissue culture dish with cover glass bottom (35 mm FluoroDish^{TM}, World Precision Instruments) lined inside with a strip of damp paper towel to maintain humidity throughout the course of the experiment. The dish was closed and sealed with a strip of parafilm, then placed in the microscope's environmental box (Life Imaging Services, Switzerland). Cells were allowed to settle for 30 min before experiment start.

2.2 Scoring

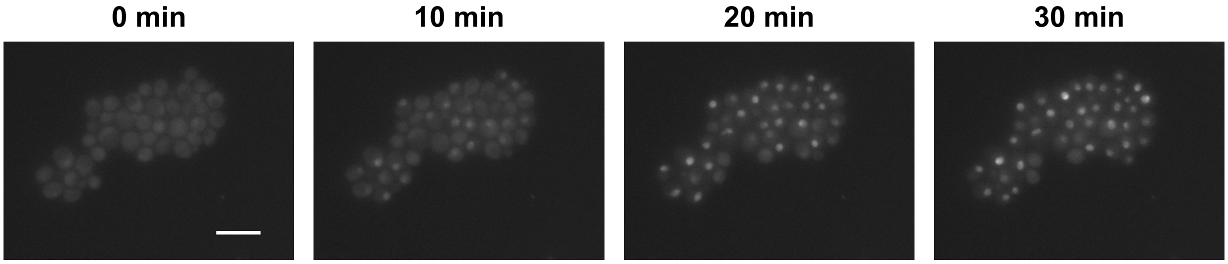

Figure 2: Representative time lapse of reporter system. Pictured are maximum projection fluorescence images for cells under constant illumination, taken from one of four preliminary dose response experiments (see Section 2.4). Scale bar is 10 µm.

We assessed induction of gene expression using a fast-acting, nuclear translocation-based reporting system [2], where higher responses corresponded to greater fluorescence in the cell nucleus. Accordingly, we defined the scoring scheme as

$$\text{score} = \frac{\text{mean fluorescence in nucleus} - \text{mean fluorescence in cytoplasm}}{\text{mean fluorescence across entire cell}}$$

such that a score of 0 indicates no difference between nuclear and cytoplasmic fluorescence.

Our experiments required us to calculate the response score automatically at each time step. The score for each automatically segmented cell was calculated for an approximated location of the nucleus, determined as follows: First, an image was formed by taking a box around the segmented cell in the maximum projection fluorescence image and converting the non-cell pixels to black. A black border 5 pixels wide was added around the image. Then the image was blurred with a Gaussian filter of standard deviation 2 pixels and the brightest pixel in the blurred image was located. This point was considered to be the center of the nucleus. We noted by observation that the nucleus was almost always 5 pixels in radius, therefore the pixels falling within a circle of radius 5 pixels around the center were presumed to belong to the nucleus and were subsequently used in the calculations of the mean fluorescence. All remaining pixels belonging to the cell were considered to belong to the cytoplasm. The score was then calculated as indicated above.

We estimated the time constant for cell response as the time to reach half the maximum score, calculated from an average score over cells. For these experiments, we sampled cells every 2 min for a step input from zero to the maximum intensity delivered during patterning experiments, and obtained a time constant of 10.9 min ($N = 63$ cells). We also calculated the time constant based on an input drop from maximum intensity to zero, and obtained a similar time constant of 10.3 min ($N = 65$ cells).

2.3 Segmentation/tracking errors

The automated imaging pipeline occasionally failed to identify cells in particular frames. In the majority of cases the system was able to recover the cell within one or two frames. In dose response experiments, cells that were not tracked for the entirety of a dose were discarded. During patterning experiments, cells that were not tracked in a frame did not receive input for the following 10 min, and scores for their corresponding patches were calculated as averages over the scores of the remaining cells. In no experiments were all cells in a patch simultaneously dropped in the same frame.

	K	Fraction Dropped Measurements
**16-patch**	0.1	0.041666667
	0.1	0.029605263
	0.1	0.049342105
	0.1	0.026864035
	0.2	0.038377193
	0.2	0.038377193
	0.2	0.091557018
	0.3	0.10252193
	0.3	0.097039474
	0.3	0.064144737
	1	0.029057018
	1	0.04879386
	1	0.090460526

	K	Fraction Dropped Measurements
**36-patch**	0.1	0.094298246
	0.1	0.041666667
	0.1	0.119152047
	0.1	0.148391813

Table 1: Fraction of all measurements that were dropped per experiment. One measurement is a single cell in a single frame.

2.4 Preliminary dose response experiments verify gradedness and independence of dose history

In order for the theory to apply, we needed to verify (a) that the magnitude of cell response increased with received light intensity; and (b) that the dose response would be independent of dose history for the duration of the final patterning experiments. By "dose history", we refer to the number, intensity, and order of doses administered to cells prior to a particular time of interest.

The following experiments were performed with an ND filter of optical density 2 (Thorlabs, 25 mm absorptive). Cells were illuminated for 10 min with uniform light, then left in the dark for 20 min before doses were administered. Cells were imaged every 10 min. Doses were administered for 40 min and the administered intensity of a dose varied for different collections of cells on the same plate simultaneously. Hereafter, we use "dose response experiment" to refer to a particular collec-

tion of cells on the same plate receiving the same series of administered doses. Two dose response experiments were conducted simultaneously per plate, with four or five doses per experiment. For a single time point, the measured projected intensity received by a cell was calculated as the average of the mean measured projected intensity across all pixels occupied by the cell.

A series of general linear models were fit to the data for each cell. The natural log of the score (response variable) was treated as a function of the agarose pad, frame, dose number (ordered by time of appearance during experiment), frames since dose start, current measured illumination intensity, and the measured illumination intensity for all frames up to the minimum experimental duration (16 frames) before the current frame. Intensities for time points before the start of the experiment were set to 0. An analysis of deviance for the full model (Table 2) suggests that the illumination history past the current illumination contributes little to the current score.

Furthermore, we determined by observation that cells had reached a quasi-steady state response before 30 min, and found that fitting a general linear model to steady-state times only (30 min and 40 min into a dose) greatly diminishes the importance of plate, frame, dose number, and frames since dose start (Table 3). Moreover, a reduced model for the steady-state score that includes only the current intensity has an AIC substantially similar to that of the full steady-state model (2675 for the reduced vs. 2622 for the full). Together, these analyses suggest that it is reasonable to consider steady-state dose response as a function of current illumination intensity alone.

2.5 Ensuring uniform illumination intensity

We calculated our intensity corrections based on the following model: If u is an $N \times N$ input image (normalized to $[0,1]$) and u_r is that image magnified to size $M \times M$, then the measured intensity y (normalized to $[0,1]$) is an $M \times M$-dimensional image given by

$$y = s(v(u_r))$$

where $s(\cdot)$ is a sigmoidal function applied identically to each pixel, and $v(\cdot)$ is a function that varies by pixel to represent the uneven illumination intensity. This suggests that, to achieve a desired y_d, an input image should be calculated as

$$u_r = v^{-1}(s^{-1}(y_d))$$

(which can be appropriately resized to obtain u).

$s^{-1}(\cdot)$ was already determined from previous experiments with the setup [3]. This correction was calculated once, as it did not appear to change between experiments. $v^{-1}(\cdot)$ was calculated before each experiment by sampling the measured intensity at a number of points spaced across the sample plane when the administered intensity was maximized. To do so, one hundred circles arranged in a grid were projected one at a time onto the slide at maximum administered intensity and the reflected images for each circle were measured. The mean measured intensity of all the pixels in a single circle was treated as a sample of the "true" illumination intensity at the point on the sample plane corresponding to the center of the circle, such that a 2D quadratic polynomial surface could be fit to all mean circle intensities in order to interpolate the illumination intensity at all points on the sample plane. These sample points were normalized to the maximum and a second 2D quadratic polynomial surface was fit to the inverse sigmoid of these normalized sample points. A target intensity value was chosen and the surface fit was renormalized relative to this intensity value to obtain a matrix V representing the factor by which bright areas were overilluminated

Variable	Df	Deviance	Resid. Df	Resid. Dev	Pr(>Chi)
NULL	NA	NA	4117	3905.828561	NA
Im0	1	1051.353764	4116	2854.474798	0
Im1	1	148.2219005	4115	2706.252897	1.65E-53
Im2	1	0.229287964	4114	2706.023609	0.544725097
Im3	1	4.35808067	4113	2701.665528	0.008275942
Im4	1	1.320218071	4112	2700.34531	0.146119572
Im5	1	0.074542198	4111	2700.270768	0.729833304
Im6	1	0.240410136	4110	2700.030358	0.535126523
Im7	1	1.161222828	4109	2698.869135	0.17286582
Im8	1	0.187482186	4108	2698.681653	0.583904657
Im9	1	2.286447549	4107	2696.395205	0.055792838
Im10	1	0.249079692	4106	2696.146126	0.527855487
Im11	1	0.596308281	4105	2695.549817	0.328685196
Im12	1	0.142415919	4104	2695.407402	0.633115034
Im13	1	0.323652973	4103	2695.083749	0.471767645
Im14	1	0.836572299	4102	2694.247176	0.24730139
Im15	1	1.060493657	4101	2693.186683	0.192713497
Im16	1	3.170352656	4100	2690.01633	0.024309053
FramesSinceDoseStart	1	106.9288382	4099	2583.087492	4.29E-39
Pad	1	13.86651829	4098	2569.220973	2.47E-06
Frame	1	0.002190109	4097	2569.218783	0.952796489
DoseNumber	1	9.159326761	4096	2560.059457	0.00012912

Table 2: General linear model of cell response score at all points in time. Model was calculated using data from four preliminary dose response experiments. ImX indicates the measured projected intensity preceding the current by X frames (0 is current frame). The AIC for this model is 9745. As expected, the time since dose start contributes a large drop in deviance, as cells did not instantly settle to a new steady state when the intensity was changed.

relative to areas of underillumination, such that the matrix $\tilde{V}$ consisting of the element-by-element inverse of V (i.e., $\left[\tilde{V}\right]_{ij} = \frac{1}{[V]_{ij}}$) was normalized to $[0, 1]$. Subsequently, inputs u_r were calculated to achieve the desired output y_d as

$$u_r = \tilde{V} \odot s^{-1}(y_d)$$

where $\odot$ indicates element-by-element multiplication.

The sigmoidal intensity correction was used for all experiments. The flattening correction procedure was carried out before experiment start on an area of the plate unoccupied by cells, and was performed for all experiments except the dose response experiments.

3 Patch-level dose response

The deterministic theory operates under the assumption that the dose response curve is identical for all cells and therefore all patches. To examine the similarities between the empirical fitted dose response curve and actual patch-level dose responses, we bootstrapped from a single dose response

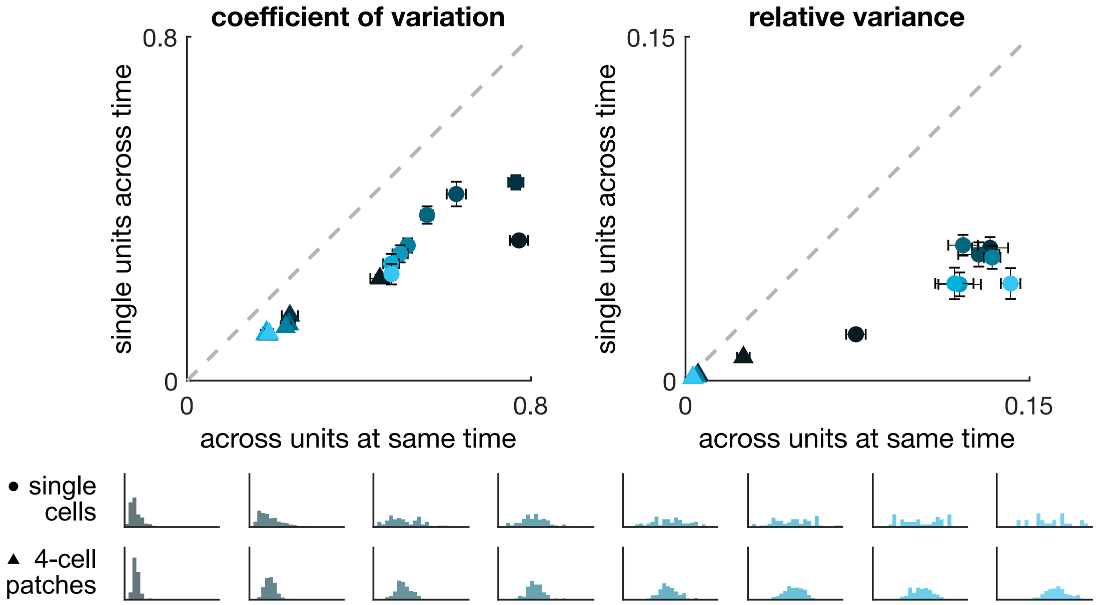

Figure 3: Cell-cell and patch-patch variability in cell response score for 4-cell patches at different illumination intensities. Plots correspond to those in Figure 3d, but for 4-cell instead of 6-cell patches. Scores were binned by projected intensity (increasing from black to light blue) to generate histograms of score distributions for given input levels (samples per bin left to right: 344, 183, 69, 108, 102, 57, 35, 26). Bin cutoff is twice the maximum projected intensity used in final patterning experiments. Histograms for 4-cell patches were generated for each bin by bootstrapping from individual cell scores within that bin for a total of 200 bootstrapped patches per intensity level. Circles indicate cell-cell variability; triangles indicate patch-patch variability. Error bars are s.e.m.

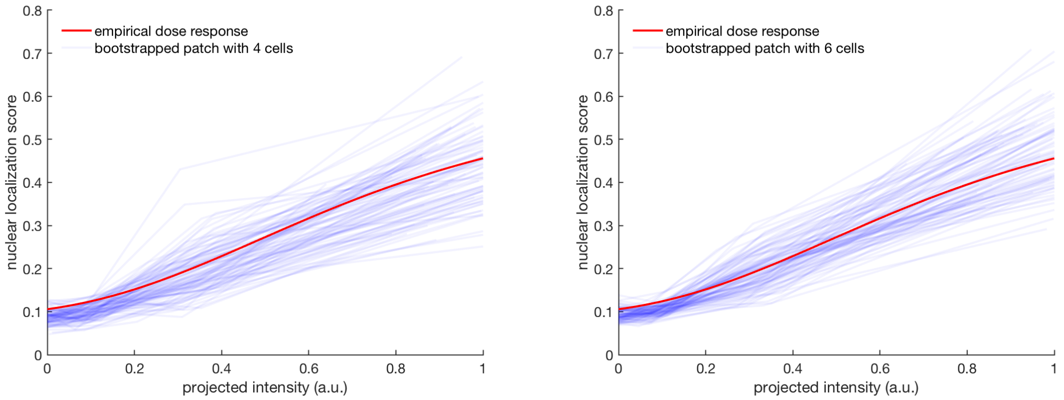

Figure 4: Patch-level dose responses approximate empirically fitted dose response. Fitted dose response (red) vs. 100 bootstrapped dose responses (blue) for patches of 4 or 6 cells, drawn from a single dose response experiment. Note that the fitted response is a curve, while the bootstrapped responses are linear interpolations over four points.

Variable	Df	Deviance	Resid. Df	Resid. Dev	Pr(>Chi)
NULL	NA	NA	1185	1140.234564	NA
Im0	1	481.0998049	1184	659.1347596	1.03E-201
Im1	1	15.65834255	1183	643.476417	4.58E-08
Im2	1	0.206888356	1182	643.2695287	0.529734222
Im3	1	1.351828641	1181	641.9177	0.108199518
Im4	1	1.058581525	1180	640.8591185	0.155179456
Im5	1	0.658939118	1179	640.2001794	0.262075464
Im6	1	0.111574008	1178	640.0886054	0.644450512
Im7	1	0.009561578	1177	640.0790438	0.892536317
Im8	1	2.783572744	1176	637.2954711	0.021164596
Im9	1	1.374292916	1175	635.9211782	0.105311673
Im10	1	3.334521299	1174	632.5866569	0.011640495
Im11	1	1.633214394	1173	630.9534425	0.077459177
Im12	1	0.519193207	1172	630.4342493	0.319493668
Im13	1	0.000278015	1171	630.4339712	0.981621402
Im14	1	3.592110318	1170	626.8418609	0.008831956
Im15	1	2.201528614	1169	624.6403323	0.040371525
Im16	1	3.868935675	1168	620.7713966	0.006577405
FramesSinceDoseStart	1	4.202474492	1167	616.5689221	0.004622429
Pad	1	0.000989428	1166	616.5679327	0.965336552
Frame	1	6.225817085	1165	610.3421156	0.000566296
DoseNumber	0	0	1165	610.3421156	NA

Table 3: General linear model of cell response score during steady state. Model was calculated using data from four preliminary dose response experiments for steady-state frames (30 and 40 min after dose start) only. ImX indicates the measured projected intensity preceding the current by X frames (0 is current frame). Note that the frames since dose start, pad, frame, and dose number are less significant in this model relative to the full model in 2. The AIC for this model is 2622.

experiment for $N = 100$ patches with 4 or 6 cells per patch. Our final setup did not permit us to construct full dose response curves for individual cells or patches, as we could administer a maximum of four doses per experiment, which is not sufficient to fit a curve with four degrees of freedom. Therefore, we visually compared a linear interpolation of bootstrapped dose responses to the administered doses with the empirical fitted dose response curve (Supplementary Figure 4). The patch-level dose responses do exhibit some level of variation (higher for higher projected intensity), which probably manifests in the experiments as systematic variation in average patch intensity. This patch-patch variation, in turn, may be the most notable contributor to the experiment-to-experiment variation in exact contrast level and overall brightness observed in our patterning system (Figure 5, Supplementary Figure 5).

4 Patterning experiments

4.1 Input adjustment time

The frequency of input adjustment was lower bounded by the amount of time it took for image acquisition and analysis (minimum ∼2 min, somewhat longer the more cells were in the field). Adjustment time was also loosely upper bounded based on the practicality that cells would eventually grow out of a monolayer, which mandated that inputs be adjusted with enough frequency for a pattern to appear within 3 or 4 hours.

Within these constraints, we chose the frequency of input adjustment to be on the same order of magnitude as the response time of the cells. Specifically, we decided to adjust inputs faster than the ∼20 or 30 minutes for individual cells to reach steady state so that we could (a) ascertain when the *full system* (not just individual cells) had reached steady state by plotting the instantaneous input/output relation during the experiment, and (b) subject the theory to a more stringent test (i.e., make sure transient patch dynamics could still affect the dynamics of the full system without nullifying predictions made from single-cell steady-state behavior alone).

4.2 Global oscillations in score at experiment start

The apparent global oscillations in score before contrast emerges in the $K = 0.1$ case (Figure 5a) may be a consequence of the fact that cell responses are delayed relative to the frequency with which administered inputs are changed. Intuitively, since all patches begin with similar scores, then during the next imaging period the inputs to all patches will also be roughly the same magnitude. Assuming the scores are initially low, then the lateral inhibition relationship ensures that inputs during the next imaging period will all be similarly high. Cell scores will continue to increase for about two periods following a period of high input, such that an intervening single period of lower input will not drastically affect cell score until about two periods later, when the input has decreased even further due to the continued rise in score from the first high input. A similar effect causes a steep drop in cell scores, restarting the cycle. Imperfect initial conditions and stochasticity ensure that the oscillations eventually decay, allowing for convergence to the contrasting steady state.

4.3 Permutation tests to verify contrast

Stochasticity introduces some difference between the mean scores of any two sets of patches regardless of whether they alternate. We conducted statistical tests to verify that the difference in mean between sets of alternating patches exceeded what would be expected for any other arbitrary grouping of patches into two equal-sized sets, i.e., that the contrast arose as a consequence of lateral inhibition separating the patches into two distinct populations of low- and high-scoring cells, rather than due to chance variation in mean scores alone for cells belonging to the same population. In particular, for each experiment we conducted permutation tests against an empirical distribution of $N = 5000$ relabelings, with the null hypothesis that scores for alternating patches were drawn from the same distribution (i.e., the partition into sets of alternating patches is arbitrary). Tests were conducted three times per experiment with results reported in Table 4. The contrast in the $K = 0.1$ case was statistically significant in all experiments, while the apparent contrast in the $K = 1$ case was statistically indistinguishable from contrast between random sets of patches. For each of $K = 0.2$ and $K = 0.3$, the majority (2 out of 3) of replicates yielded results consistent with theoretical notions of mono- and bistability at a p-value of 0.05.

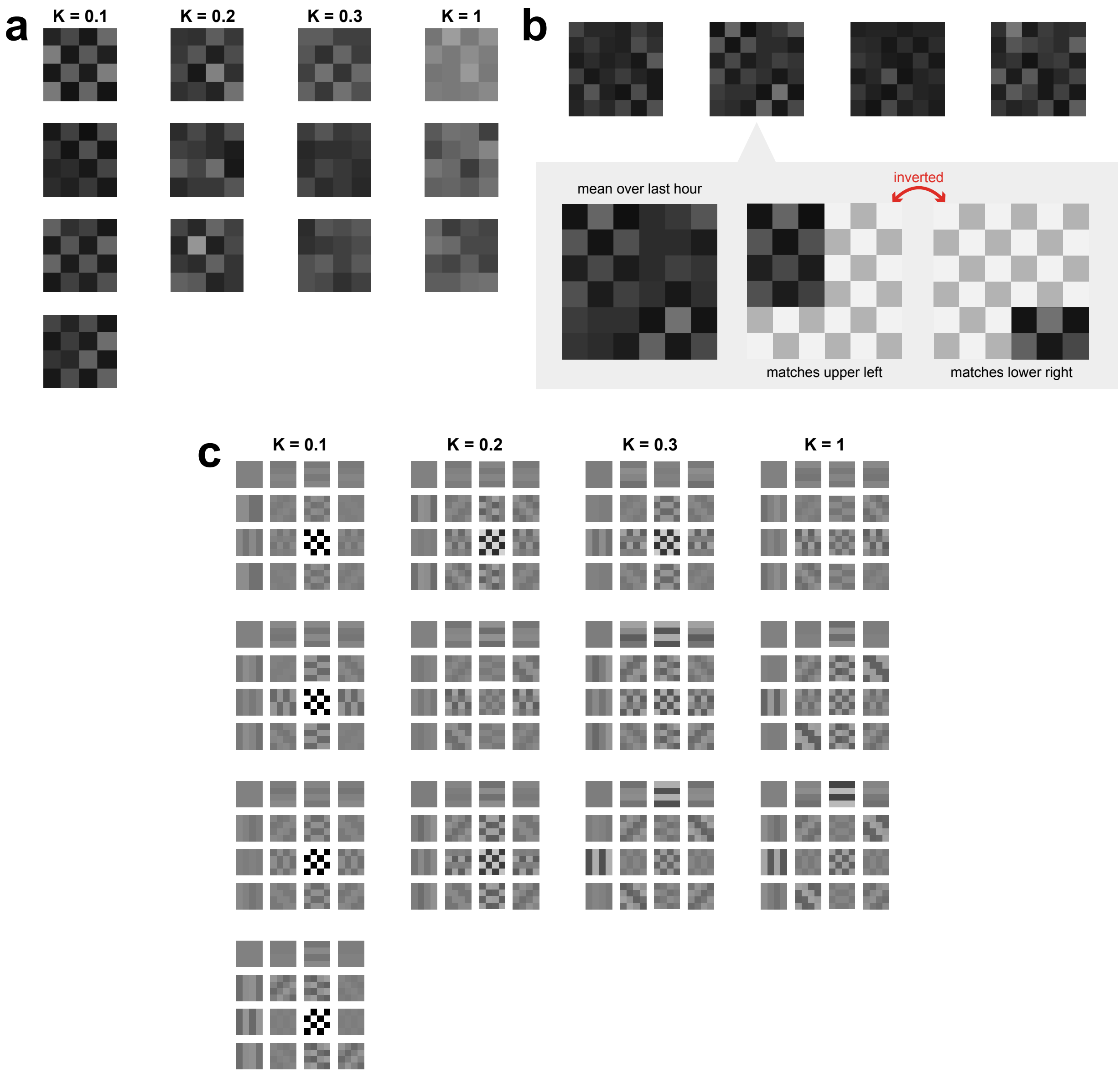

Figure 5: Checkerboards observed in all experiments. Visualizations are rendered from scores averaged over the last hour for (a) 16-patch and (b) 36-patch ($K = 0.1$) patterning experiments. Although the 36-patch experiments did not achieve global checkerboard patterning, one experiment showed persistent local patterning in which two small checkerboards appeared in opposing corners. The local patterns were inverted relative to each other and did not resolve before the end of the experiment. (c) 2D Fourier transforms conducted on the checkerboard averages from (1) reveal that the greatest weight is given to the highest-contrast spatial mode in all 4 experiments with $K = 0.1$, 2 out of 3 experiments with $K = 0.2$, 1 out of 3 experiments with $K = 0.3$, and none of the 3 experiments with $K = 1$. Pictured are the spectral components (horizontal frequency increasing top to bottom, vertical frequency increasing left to right) that contribute to last-hour checkerboards for each experiment. The intensity of each component indicates its weighting relative to other components in the same experiment. Note also the relatively higher weighting for lower-frequency spatial modes in the $K = 0.3$ and $K = 1$ cases relative to the $K = 0.1$ and $K = 0.2$ cases. Source data are provided as a Source Data file.

K	trial1	trial2	trial3
0.1	0	0.0002	0
0.1	0	0	0
0.1	0	0	0
0.1	0	0	0
0.2	0.0006	0.0008	0.0008
0.2	0.4422	0.4348	0.4356
0.2	0.0234	0.0226	0.017
0.3	0.0158	0.0156	0.0164
0.3	0.1262	0.1282	0.1322
0.3	0.146	0.1348	0.1388
1	0.6516	0.6444	0.645
1	0.4108	0.4116	0.4018
1	0.1966	0.1942	0.2144

Table 4: Differences in mean score between alternating sets of patches exceeded differences between random sets of patches. Two-tailed permutation tests for difference in mean score averaged across the last hour between sets of alternating patches, conducted against empirical distributions constructed from N = 5000 relabelings for each experiment individually. The empirical distributions of difference in means between relabeled sets of patches were randomly drawn three times per experiment and the resulting p-value calculated for the alternating patch allocation. Tests were performed against the null hypothesis that scores were drawn from the same distribution for all patches. For $K = 0.1$, the null hypothesis was rejected at a p-value of 0.0002 in all four experiments. For $K = 1$, the null hypothesis failed to be rejected at any reasonable p-value. Results were less clear for values close to the critical point, for which one experiment with $K = 0.2$ (theoretically bistable) failed to reject at any reasonable p-value, and one experiment with $K = 0.3$ (theoretically monostable) rejected at a p-value of 0.02.

4.4 Temporal variability in score

Although variability in administered intensity decreased from the first to the last experimental hour in 16-patch experiments, temporal variability in individual patch scores remained nearly the same throughout the experiment (Supplementary Figure 6a). The result is not surprising if cells were comparably variable regardless of input intensity, in which case convergence of the mean score need not imply reduction in temporal variability. The administered intensity may also appear less variable since it was calculated based on an average over the scores of 4 neighbors, which at a given time step would have a variance $\frac{1}{16}$ that of an individual patch.

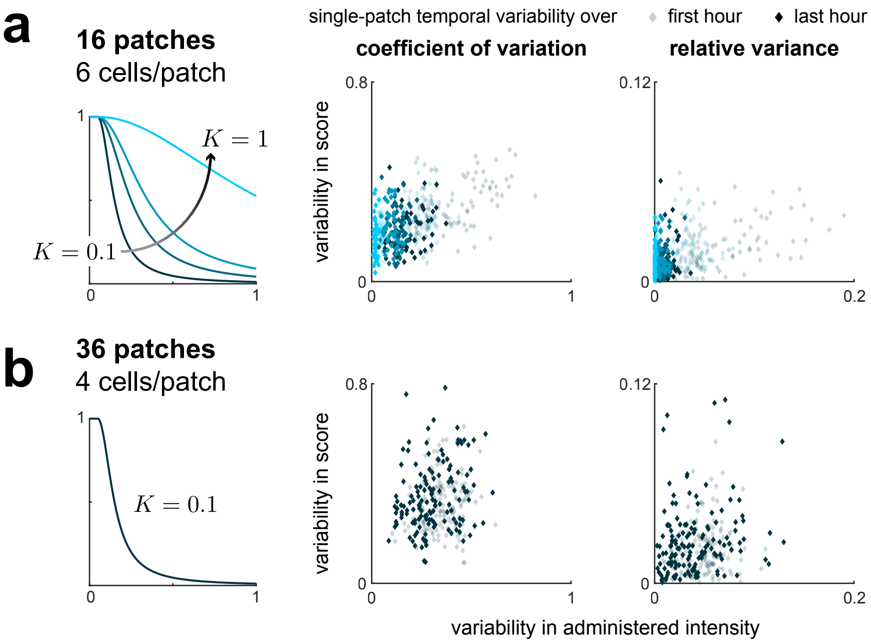

Figure 6: Temporal variability in administered intensity during first or last experimental hour. (a) Temporal variability in score and administered intensity to individual patches decreased from the first to the last experimental hour for all 16-patch patterning experiments, while temporal variability in score remained relatively constant. (b) In four experiments with 36 patches and 4 cells per patch, temporal variability in administered intensity and score for individual patches remained equally high throughout the experiment duration (3 hr), suggesting steady state was not reached in that time. Source data are provided as a Source Data file.

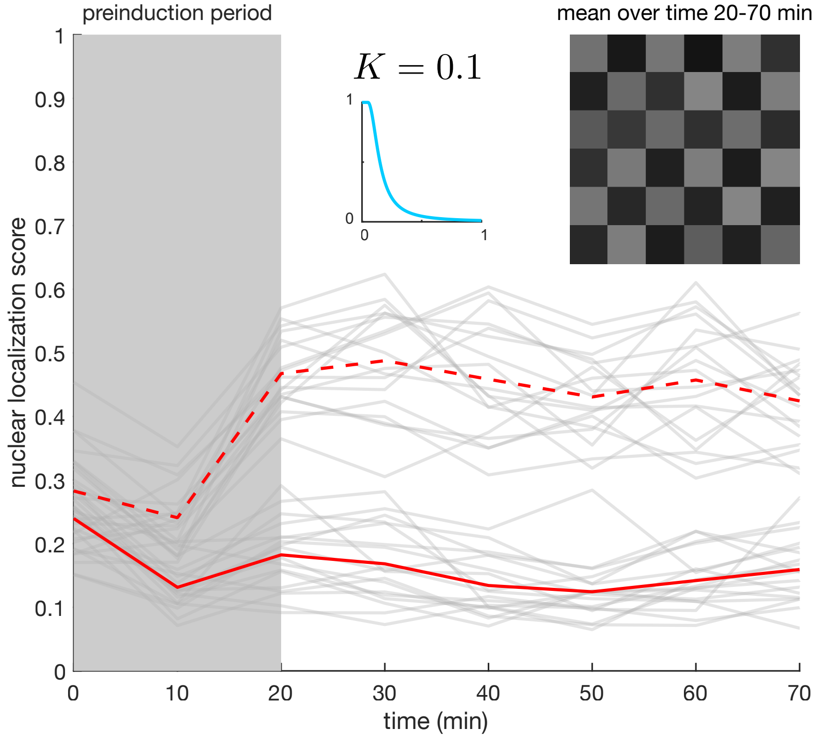

Figure 7: Control experiment verifying persistence of checkerboard pattern in full lateral inhibition system with 36 patchs, 4 cells/patch. Patches were preinduced for 20 min to display a checkerboard pattern. From 20 to 70 min, the system was run in closed-loop with lateral inhibition signaling relation $K = 0.1$. Pictured is the time course for individual patch scores (gray) with averages over sets of alternating patches in red (as in Figure 4). Board is visualization for individual patch scores averaged over the last 50 min.

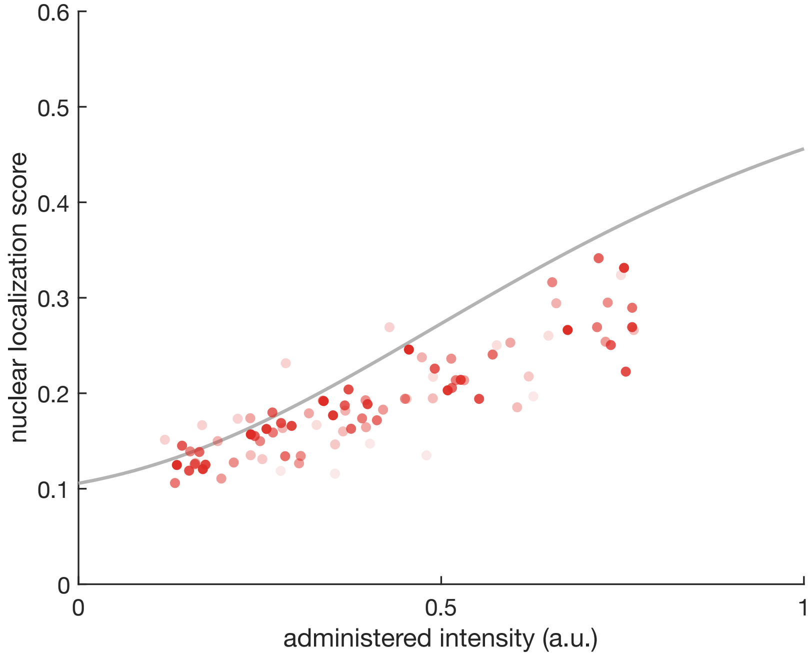

Figure 8: Administered input vs. measured patch score over time in the 36-patch experiments. The scatterplot does not show convergence to the steady-state dose response curve in 3 hr, unlike in the 16-patch case (Figure 5). Points are quantiles across $N = 4$ experiments with $K = 0.1$ for single time points, with more opaque (darker) points corresponding to later times. Source data are provided as a Source Data file.

References

[1] Angeli, D., Ferrell, J., James E. & Sontag, E. D. Detection of multistability, bifurcations, and hysteresis in a large class of biological positive-feedback systems. *Proc. Natl. Acad. Sci. U.S.A.* **101**, 1822–1827 (2004).

[2] Aymoz, D., Wosika, V., Durandau, E. & Pelet, S. Real-time quantification of protein expression at the single-cell level via dynamic protein synthesis translocation reporters. *Nat. Commun.* **7:11304** (2016).

[3] Rullan, M., Benzinger, D., Schmidt, G. W., Milias-Argeitis, A. & Khammash, M. An optogenetic platform for real-time, single-cell interrogation of stochastic transcriptional regulation. *Mol. Cell* **70**, 745–756 (2018).
