# Supplementary material for: Cell-in-the-loop pattern formation with optogenetically emulated cell-to-cell signaling
Source: Nat Commun. 2020 Mar 13;11:1355. doi: 10.1038/s41467-020-15166-3 (PMC7069979; doi:10.1038/s41467-020-15166-3)
Supplement: Supplementary file 3 — Reporting Summary [file 41467_2020_15166_MOESM3_ESM.pdf]

nature research

Corresponding author(s): Melinda Liu Perkins
Mustafa Khammash

Last updated by author(s): Feb 5, 2020

Reporting Summary

Nature Research wishes to improve the reproducibility of the work that we publish. This form provides structure for consistency and transparency in reporting. For further information on Nature Research policies, see Authors & Referees and the Editorial Policy Checklist .

Statistics

For all statistical analyses, confirm that the following items are present in the figure legend, table legend, main text, or Methods section.

n/a	Confirmed	
☐	☒	The exact sample size (n) for each experimental group/condition, given as a discrete number and unit of measurement
☐	☒	A statement on whether measurements were taken from distinct samples or whether the same sample was measured repeatedly
☐	☒	The statistical test(s) used AND whether they are one- or two-sided<br>*Only common tests should be described solely by name; describe more complex techniques in the Methods section.*
☐	☒	A description of all covariates tested
☐	☒	A description of any assumptions or corrections, such as tests of normality and adjustment for multiple comparisons
☐	☒	A full description of the statistical parameters including central tendency (e.g. means) or other basic estimates (e.g. regression coefficient) AND variation (e.g. standard deviation) or associated estimates of uncertainty (e.g. confidence intervals)
☐	☒	For null hypothesis testing, the test statistic (e.g. F, t, r) with confidence intervals, effect sizes, degrees of freedom and P value noted<br>*Give P values as exact values whenever suitable.*
☒	☐	For Bayesian analysis, information on the choice of priors and Markov chain Monte Carlo settings
☒	☐	For hierarchical and complex designs, identification of the appropriate level for tests and full reporting of outcomes
☒	☐	Estimates of effect sizes (e.g. Cohen's d, Pearson's r), indicating how they were calculated

Our web collection on statistics for biologists contains articles on many of the points above.

Software and code

Policy information about availability of computer code

Data collection	Optogenetic inputs and images were acquired using YouScope (open-source microscope software) in combination with custom MATLAB scripts adapted from Rullan and Benzinger et al. (2018). Segmentation and tracking was performed using software tools developed by Rullan and Benzinger et al. (2018) based on Dimopoulos et al. (2014) and Ricicova et al. (2013).
Data analysis	Data analysis was performed using custom Matlab (MathWorks) scripts.

For manuscripts utilizing custom algorithms or software that are central to the research but not yet described in published literature, software must be made available to editors/reviewers. We strongly encourage code deposition in a community repository (e.g. GitHub). See the Nature Research guidelines for submitting code & software for further information.

Data

Policy information about availability of data

All manuscripts must include a data availability statement. This statement should provide the following information, where applicable:

- Accession codes, unique identifiers, or web links for publicly available datasets
- A list of figures that have associated raw data
- A description of any restrictions on data availability

The cell score data underlying Figures 3c and 5, as well as Supplementary Figures 5, 6, and 7, are available in the Source Data file. Remaining data, plasmids, and strains are available upon request.

Field-specific reporting

Please select the one below that is the best fit for your research. If you are not sure, read the appropriate sections before making your selection.

Life sciences study design

All studies must disclose on these points even when the disclosure is negative.

Sample size	Pooled data from dose response experiments yielded n = 180 or 181 measurements per quantile, which is greater than the n = 170 required to estimate the mean per quantile with 99% confidence, expected population SD 0.5, margin of error 0.1 (t-distribution to estimate sample size). Replicates for patterning experiments were chosen at minimum n = 3 following standard practice (e.g., Sekine et al. 2018, Benzinger and Khammash 2018).
Data exclusions	No data were excluded.
Replication	All attempts at replication were successful. Each experiment was repeated a minimum of 3 times as indicated in figure legends.
Randomization	Cells were randomly allocated to experiments as well as to patches within experiments.
Blinding	Researchers were not blinded during the study because data processing and analysis were automated. Experiments were performed with cells randomly sampled from culture with no selection bias.

Reporting for specific materials, systems and methods

We require information from authors about some types of materials, experimental systems and methods used in many studies. Here, indicate whether each material, system or method listed is relevant to your study. If you are not sure if a list item applies to your research, read the appropriate section before selecting a response.

Materials & experimental systems

n/a	Involved in the study	
☒	☐	Antibodies
☒	☐	Eukaryotic cell lines
☒	☐	Palaeontology
☒	☐	Animals and other organisms
☒	☐	Human research participants
☒	☐	Clinical data

Methods

n/a	Involved in the study	
☒	☐	ChIP-seq
☒	☐	Flow cytometry
☒	☐	MRI-based neuroimaging
